# First Detection and Genetic Characterization of New Equine Parvovirus Species Circulating among Horses in Korea

**DOI:** 10.3390/vetsci8110268

**Published:** 2021-11-07

**Authors:** Jungho Yoon, Taemook Park, Ahram Kim, Heeeun Song, Byung-Joo Park, Hee-Seop Ahn, Hyeon-Jeong Go, Dong-Hwi Kim, Joong-Bok Lee, Seung-Yong Park, Chang-Seon Song, Sang-Won Lee, In-Soo Choi

**Affiliations:** 1 Equine Clinic, Jeju Stud Farm, Korea Racing Authority, Jeju 63346, Korea; junghoy11@gmail.com (J.Y.); taemook7@gmail.com (T.P.); aidia0207@naver.com (A.K.); vetsongheen@gmail.com (H.S.); 2 Department of Infectious Diseases, College of Veterinary Medicine, Konkuk University, Seoul 05029, Korea; twilightsd@naver.com (B.-J.P.); heesuob2@naver.com (H.-S.A.); misilseju@naver.com (H.-J.G.); opeean0@naver.com (D.-H.K.); virus@konkuk.ac.kr (J.-B.L.); paseyo@konkuk.ac.kr (S.-Y.P.); songcs@konkuk.ac.kr (C.-S.S.); odssey@konkuk.ac.kr (S.-W.L.)

**Keywords:** equine parvovirus, equine parvovirus-CSF, eqcopivirus, horses, prevalence, risk factor

## Abstract

Equine parvovirus-cerebrospinal fluid (EqPV-CSF) and eqcopivirus (EqCoPV) are new parvovirus species (EqPVs) identified from various tissues (CSF, blood, and respiratory swabs) in horses with neurologic and respiratory diseases. In this study, we described the prevalence rate of EqPV-CSF and EqCoPV in 133 and 77 serum and fecal samples, respectively, using polymerase chain reaction. Further, we analyzed the potential risk factors for infection. We calculated the nucleotide and amino acid similarity and constructed phylogenetic trees. There was a moderate-to-high prevalence rate (EqPV-CSF: 3.8%; EqCoPV 9.8%) of each virus in serum; moreover, age, country of foaling, and clinical colic signs were significantly associated with the EqPVs infection. The newly identified EqPV-CSF/EqCoPV genomes had high nucleotide and amino acid identities with previously isolated strains in the USA. In phylogenetic analysis, they clustered and formed a new subgroup in the genus *Copiparvovirus*. To our knowledge, this is the first field epidemiologic study on EqPV-CSF and EqCoPV using both serum and fecal samples. Our findings demonstrate the risk factors for infection and could facilitate the development of disease prevention strategies.

## 1. Introduction

Since the first isolation of the equine parvovirus in 1985, three equine parvovirus species (EqPVs), which belong to the genus *Copiparvovirus* and family *Parvoviridae*, were identified; namely, equine parvovirus-cerebrospinal fluid (EqPV-CSF, 2015), equine parvovirus-hepatitis (EqPV-H, 2018), and Eqcopivirus (EqCoPV, 2019) [1,2,3,4]. Except for EqPV-H, which is an etiologic agent for equine serum hepatitis [4,5,6,7,8,9,10,11,12,13,14,15], the other two species have not been well investigated.

EqPV-CSF was first isolated in 2015 from cerebrospinal fluid (CSF) in a horse with neurological signs and lymphocytic pleocytosis in the USA [2]. Subsequently, a Chinese study reported a high prevalence of EqPV-CSF nucleotide (25.3%, 39/152) in sera obtained from imported horses in Western Europe but not local horse breeds [16]. In 2019, EqCoPV, which is another novel equine parvovirus, was isolated in the USA from horse plasma, CSF, and respiratory swabs from 14 horses [1]. However, the relationship between EqPV-CSF/EqCoPV and clinical disease in horses remains unclear given the limited sample sizes and missing evidence [1,16].

This is the first study to report the prevalence and genetic history of EqPV-CSF and EqCoPV among horses in Korea using serum and fecal samples. Studies have demonstrated that EqPV-CSF circulates in imported horses or offspring of imported dams. Unlike EqPV-CSF, EqCoPV has endemic characteristics and has been detected in local and foreign breed horses with a high prevalence rate. Especially, both viruses were related with clinical colic cases under statical significance (*p* < 0.05). Our findings could expand the current knowledge regarding EqPV-CSF/EqCoPV and facilitate the control of epidemiologic risk factors. 

## 2. Material and Methods

### 2.1. Sample Collection

We collected 133 serum and 77 fecal samples from 199 horses (both serum and fecal samples were collected from 11 horses) in Jeju Province, which is a major horse-breeding region in Korea. Specifically, 88 horses presented for medical treatment with clinical signs (Supplementary Table S2), 111 horses were healthy. Blood was collected using a venipuncture into Vacutainer^®^ SST^TM^ blood collection tubes (Becton-Dickinson, Franklin Lakes, NJ, USA), with separation of sera after centrifugation. Fecal sampling was performed per rectum or shortly after defecation for each horse using sterile gloves [17]. Subsequently, 1 g of feces and 10 mL of phosphate-buffered saline were vortexed, followed by harvesting of fecal supernatants after centrifugation at 3000× *g* for 30 min. All serum and fecal supernatants were stored at −70 °C until further analysis. The animal protocols were approved by the Institutional Animal Care and Use Committee of Korea Racing Authority (KRA IACUC-2106-AEC-2106).

### 2.2. Virus Detection and Complete Coding Sequence Assembly

Viral nucleic acids from serum or feces were extracted with Patho Gene-Spin™ DNA/RNA kit (Intron Biotechnology, Seongnam, Korea) using 150 μL of serum or fecal supernatants, followed by elution in 30 μL of elution buffer. The specific sequences of EqPV-CSF and EqCoPV were detected through polymerase chain reaction (PCR) using the Maxime™ PCR PreMix kit (Intron Biotechnology, Seongnam, Korea). We used reported primers targeting nonstructural (NS) genes of EqPV-CSF [16], as well as a newly designed primer pair (forward: 5′-GTG GAC AGC CGA AGA GTG GA-3′ and reverse: 5′-AGT CAC TCG GCC ATG GTG TT-3′) for detecting viral particle (VP) genes of EqCoPV based on GenBank registered sequences [1]. PCR was initiated at 95 °C for 5 min followed by 40 denaturation cycles at 95 °C for 30 s, annealing at 50 °C for 30 s, and extension at 72 °C for 1 min. The final extension was performed at 72 °C for 10 min. PCR products were analyzed through 1.5% agarose gel electrophoresis. Correct-sized PCR products were purified using MEGAquick-spin Plus DNA Purification Kit (Intron Biotechnology, Seongnam, Korea), followed by Sanger sequencing (Cosmo Genetech, Seoul, Korea).

We generated 2 complete viral coding sequences (CDS) of each EqPV-CSF, and EqCoPV isolate using the primer walking method [18]. Supplementary Table S1 lists the primer sets designed for the complete CDS and PCR condition. The sequences were assembled using SeqMan software (DNASTAR, Madison, WI, USA).

We deposited all sequenced genes of EqPV-CSF (1 complete CDS and 4 partial NS genes) and EqCoPV (1 complete CDS and 12 partial VP genes) into the GenBank database (accession numbers OK422845 to OK422862). 

### 2.3. Phylogenetic Tree and Similarity Assessment

To evaluate the evolutionary history of the newly isolated EqPV-CSF and EqCoPV, nucleotide sequences were aligned using the MUSCLE algorithm, and phylogenetic trees were constructed with Molecular Evolutionary Genetics Analysis (MEGA) software version X (maximum likelihood method, bootstrapping value = 500) [19]. Substitution models were selected using the Best DNA/Protein model finding option of MEGA X.

We calculated the genetic distances between isolated Korean parvovirus strains and other genus *Copiparvovirus* members to determine the similarity of the amino acid and nucleotide sequences. Similarly, sequences were aligned using the MUSCLE algorithm, and *p*-distances were calculated using MEGA X.

### 2.4. Statistical Analysis

We performed statistical analyses to determine associated factors for the incidence of EqPV-CSF or EqCoPV. We performed the binomial logistic regression test (age) or chi-square test (country of foaling, horse purpose, breed, and colic sign). We reported the *p*-value, odds ratio (OR), and confidence interval of the OR. Statistical significance was set at *p* < 0.05. All statistical analyses were performed in Jamovi software (The Jamovi Project 2021, version 1.6.23).

## 3. Results

### 3.1. Prevalence of EqPV-CSF and EqCoPV

We tested 133 serum and 77 fecal samples for EqPV-CSF and EqCoPV DNA. For the serum samples, 5 (3.8%) and 13 (9.8%) horses were positive for EqPV-CSF and EqCoPV DNA, respectively (Table 1). One horse was co-infected with both viruses (Table 2). Fecal samples lacked the DNA of both viruses. Among the five horses positive for EqPV-CSF, three were imported from the USA (two breeding horses) and Australia (one riding horse), while the remaining two horses were born in Korea as offspring of imported dams. Additionally, the imported horses showed a higher prevalence rate (3/25, 12%) of EqPV-CSF DNA than Korean horses (2/108, 1.9%) (Table 1). Unlike EqPV-CSF, EqCoPV showed a high prevalence rate of viral DNA in both Korean (10/108, 9.3%) and imported horses (3/25, 12%) (Table 1). EqCoPV was widely distributed among horses regardless of the breed, age, purpose, and country of foaling (Table 1 and Table 2). In the clinical symptom analysis on 17 positive horses for EqPV-CSF or EqCoPV, 7 horses showed colic signs, 1 horse had laminitis, 1 horse was cachexia, and the others were healthy (Table 2).

### 3.2. Analysis of Risk Factors for Virus Infection

To determine potential risk factors for EqPV-CSF and EqCoPV infection, we analyzed the relationship among potential risk factors (age, country of foaling, horse purpose, breed, and clinical disease). The logistic regression model revealed a significant impact of age on EqPV-CSF, with older horses showing an increased infection chance (*p* < 0.05) (Table 3). Further, a chi-square test of potential risk factors (country of foaling, horse purpose, and clinical disease) revealed a significant relationship between EqPV-CSF infection and country of foaling (*p* < 0.05) (Table 3). Especially, both viruses are closely related to the horses with clinical colic signs (*p* < 0.05) (Table 3).

### 3.3. Phylogenetic Analysis and Sequence Similarity

**Figure 1:**
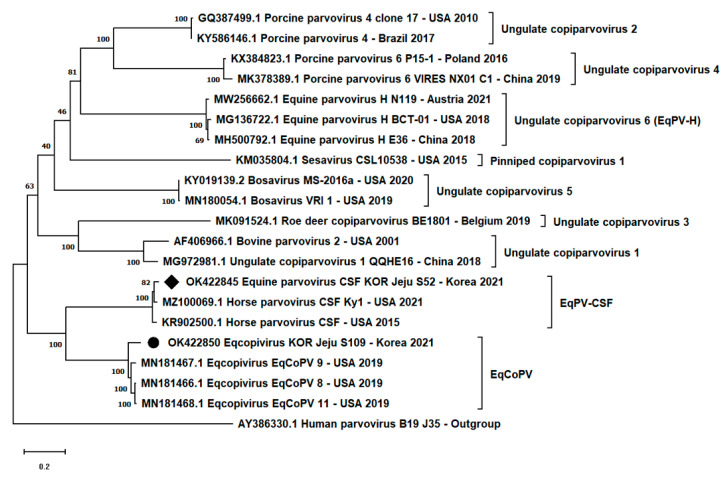
Phylogenetic analysis based on complete nucleotide sequences of genus *Copiparvovirus*. Korean isolates (EqPV-CSF: black diamond; EqCoPV: black circle) were analyzed with other members of the genus *Copiparvovirus*. GenBank accession number, country, and year of registration are labeled. Human parvovirus B19 (AY386330.1) was used as an outgroup.

We aligned the newly assembled CDS of each virus with other representative viruses in the genus *Copiparvovirus* and generated a maximum-likelihood tree. Each isolate of EqPV-CSF and EqCoPV was clustered with the previously identified strains (Figure 1). There were close relationships between the two EqPV-CSF and EqCoPV groups, which formed a distinct subgroup from the other copiparvovirus species in genus *Copiparvovirus* (Figure 1).

For analysis of the nucleotide and amino acid sequence identity, we compared the NS gene of each isolated virus with the representative virus in the genus *Copiparvovirus*. The NS gene of the isolated EqPV-CSF shared 97.9%, 56.5–58.0%, and 44.9–50.1% nucleotide identities with the previously identified EqPV-CSF strain, EqCoPV strains, and other copiparvoviruses, respectively, while the corresponding values for amino acid identities were 99.3%, 40.3–40.9%, and 25.0–30.4%. The identified NS gene of EqCoPV showed 92.1–92.4%, 57.3%, and 43.1–48.3% nucleotide identities with the previously identified EqCoPV strains, EqPV-CSF strain, and other copiparvoviruses, respectively, while the corresponding values for amino acid identities were 95.0–95.2%, 40.5%, and 25.1–32.1% (Table 4). Supplementary Figures S1 and S2, show phylogenetic trees of the partial NS genes of EqPV-CSF (four sequences, 872 bp) and partial VP genes of EqCoPV (12 sequences, 479 bp).

## 4. Discussion

Given the recent advances in the unbiased deep sequencing technique, there has been widespread distribution and active transmission of EqPVs [1,2,5,16,20,21]. However, the clinical importance of EqPVs infections is underestimated since infections with EqPVs are frequently asymptomatic [1,4,6,10,22,23,24]. Nonetheless, as shown by EqPV-H, which has become the major etiological agent for equine serum hepatitis within the last 3 years [5,6,7,8,11,13,14,15,25], there is a prevalent risk of EqPVs. Therefore, there is a need for studies regarding the little-known EqPVs to mitigate future losses. This study reported the surveillance data of EqPV-CSF and EqCoPV using both serum and fecal samples. Additionally, we identified potential risk factors for infection with both viruses. Finally, we determined the genetic and epidemiological characteristics of the isolated viruses. To our knowledge, this is the first field epidemiological study of both newly identified viruses.

As we speculated, we observed a moderate-to-high prevalence rate of EqPV-CSF (5/133, 3.8%) and EqCoPV (13/133, 9.8%) in serum (Table 1). A previous field study regarding EqPV-CSF in China reported a very high prevalence of EqPV-CSF DNA (39/152, 25.3%) in thoroughbred horses imported from Western Europe. However, the virus was not detected in local horses (0/127) [16]. This exotic characteristic of EqPV-CSF is consistent with our findings. Specifically, the EqPV-CSF prevalence rate was much higher in imported horses (3/25, 12%) than in horses born in Korea (2/108, 1.9%), with these two local horses being offspring of imported dams (Table 1 and Table 2). Contrastingly, the EqCoPV was endemic, with high prevalence rates of EqCoPV DNA in both imported (3/25, 12%) and local (10/108, 9.3%) horses (Table 1). However, the numbers of total sample and positive horses are relatively small and currently we are cautious about concluding their characteristics as dependable result.

We performed further analysis to determine associated factors for EqPV-CSF and EqCoPV. In this analysis, we used age, country of foaling, horse purpose, breed, and presence of clinical disease as predictors. We found that age and country of foaling had a close relationship with EqPV-CSF infection (Table 3). The EqPV-CSF infection increased with age; moreover, imported horses had a significantly higher risk of infection (*p* < 0.05) (Table 3). These findings demonstrate the exotic characteristics of EqPV-CSF (country of foaling). Moreover, they showed a similar infectivity pattern (age) with another equine parvovirus species, EqPV-H, which is most frequently detected in old horses [10]. As described above, the epidemiological data still have limitations on numbers of samples for putative risk factor analysis. Also, there is possibility that other intertwined factors affected the results. Our results are only first indications, and thus, additional detailed study is necessary to support our results.

In the previous studies, the correlation of clinical disease with EqPV-CSF or EqCoPV infection remains unclear [1,16,20]. Given the frequent asymptomatic infections of EqPVs [5] and increasing threats from exotic viruses [25,26,27,28], a detailed investigation of clinical signs and their epidemiology is warranted. As part of the attempt to find clinical relevance, we sorted the clinical diseases of the infected horses and then analyzed the disease association with the virus infection. The result revealed significant relations between colic signs and EqPVs infection. This is the first field evidence providing the clinical relatedness of the two, EqPV-CSF and EqCoPV, viruses. However, it does not mean that EqPV-CSF or EqCoPV causes clinical colic, or vice versa, because we still cannot verify the causal relationship between colic and virus infection. In this point, there is a need for a wide range of investigations for clinical correlation of EqPV-CSF and EqCoPV.

The tissue tropism and transmission routes of EqPV-CSF and EqCoPV remain unclear. As demonstrated in an experimental study on another equine parvovirus species, EqPV-H [6], EqPV-CSF, and EqCoPV could have tropism to various tissues. Specifically, they have been detected in several types of tissue samples (EqPV-CSF: CSF, plasma and respiratory swab; EqCoPV: plasma, CSF, respiratory swabs, lung, spleen, and colon contents); however, the studies included small sample sizes [1,2,16,20]. Moreover, a relatively large-scale study on the prevalence of EqPV-CSF in China did not detect EqPV-CSF DNA in respiratory swabs and feces, even though it showed a high prevalence in serum samples [16]. In our study, both viruses were not confirmed in feces. Currently, we cannot determine the route of viral shedding, tissue tropism, and infectivity given the limited information. Therefore, there is a need for further studies regarding their epidemiology and clinicopathology.

In the phylogenetic tree analyses, the newly isolated Korean EqPV-CSF and EqCoPV strains showed a close relationship with previously reported strains in the USA. Specifically, EqPV-CSF and EqCoPV were divided from a common ancestor and formed a distinct subgroup from other copiparvovirus species. This evolutionary characteristic is consistent with the results of sequence similarity analysis in the NS gene of each virus. EqPV-CSF and EqCoPV showed relatively higher nucleotide and amino acid similarity rates with each other than with other species in genus *Copiparvovirus*. The taxonomy of EqPV-CSF and EqCoPV remains to be established [29,30]. Moreover, our findings provide information for establishing the specific taxonomy of each virus.

To our knowledge, this is the first field study regarding the prevalence, associated factors, genetic characteristics, and evolutionary history of EqPV-CSF and EqCoPV infections. Our findings expand the current knowledge in the field basis and could inform the transmission and clinical relevance of each virus to some degree. However, there is a need for further studies regarding infectivity, pathogenicity, and transmission as potential risk factors in global horse husbandry.

## Figures and Tables

**Table 1:** Detailed information regarding the purpose, breed, country of foaling, age, and prevalence of EqPV-CSF/EqCoPV in serum among horses in Korea.

	Purpose	Breed			Average Age ^b^ (Range)	Country of Foaling			Prevalence
		TB	Jeju Horse	Others		Korea	U.S.	Others	
EqPV-CSF ^a^	Racing	2/32 (6.3%)	0/52 (0%)	0/8 (0%)	3.4 (0–11)	2/91 (2.2%)	0/1 (0%)	–	2/92 (2.2%)
	Breeding	2/23 (8.7%)	–		13.2 (7–22)	0/6 (0%)	2/17 (11.8%)	–	2/23 (8.7%)
	Riding	0/6 (0%)	0/1 (0%)	1/11 (9.1%)	8.8 (2–18)	0/11 (0%)	0/1 (0%)	1/6 (16.7%)	1/18 (5.6%)
	Total	4/61 (6.6%)	0/53 (0%)	1/19 (5.3%)	5.8 (0–22)	2/108 (1.9%)	2/19 (10.5%)	1/6 (16.7%)	5/133 (3.8%)
EqCoPV ^a^	Racing	3/32 (9.4%)	6/52 (11.5%)	1/8 (12.5%)	3.4 (0–11)	10/91 (11.0%)	0/1 (0%)	–	10/92 (10.9%)
	Breeding	2/23 (8.7%)	–	–	13.2 (7–22)	0/6 (0%)	2/17 (11.8%)	–	2/23 (8.7%)
	Riding	0/6 (0%)	0/1 (0%)	1/11 (9.1%)	8.8 (2–18)	0/11 (0%)	0/1 (0%)	1/6 (16.7%)	1/18 (5.6%)
	Total	5/61 (8.2%)	6/53 (11.3%)	2/19 (10.5%)	5.8 (0–22)	10/108 (9.3%)	2/19 (10.5%)	1/6 (16.7%)	13/133 (9.8%)

^a^ Notation order: Number of positive samples/total samples; ^b^ Year: Age at the samples were collected; TB: Thoroughbred.

**Table 2:** Information regarding equine serum samples containing EqPV-CSF and EqCoPV.

ID	Country of Foaling	Age ^a^	Breed	Purpose	Chief Complaint	EqPV-CSF	EqCoPV
1	USA	17	Thoroughbred	Breeding	Laminitis	+	
5	USA	12	Thoroughbred	Breeding	Healthy		+
11	USA	19	Thoroughbred	Breeding	Healthy	+	
23	Korea	8	Jeju-horse	Racing	Healthy		+
31	Korea	5	Jeju-horse	Racing	Healthy		+
38	Australia	18	Warm blood	Riding	Colic	+	+
52	Korea	2	Thoroughbred	Racing	Colic	+	
58	Korea	6	Jeju-horse	Racing	Healthy		+
62	Korea	7	Jeju-horse	Racing	Healthy		+
75	Korea	0	Thoroughbred	Racing	Colic		+
84	Korea	9	Jeju-horse	Racing	Healthy		+
88	Korea	3	Mixed	Racing	Healthy		+
97	Korea	9	Jeju-horse	Racing	Cachexia		+
102	Korea	0	Thoroughbred	Racing	Colic		+
109	Korea	0	Thoroughbred	Racing	Colic		+
111	USA	10	Thoroughbred	Breeding	Colic		+
133	Korea	2	Thoroughbred	Racing	Colic	+	

^a^ Year, Age at the samples were collected; EqPV-CSF, equine parvovirus-cerebrospinal fluid; EqCoPV, eqcopivirus.

**Table 3:** Statistical analysis of potential risk factors for equine parvovirus infection.

	Factors	*p*-Value	Odds Ratio	95% Confidence Interval	
				Lower	Upper
EqPV-CSF	Age	0.016 *	1.20922	1.03606	1.4113
	Country of foaling-Korea	0.016 *	0.138	0.0218	0.877
	Clinical disease—Colic	0.008 *	8.61	1.35	55.0
	Purpose-Racing	0.150	0.281	0.0452	1.75
	Purpose-Breeding	0.171	3.4	0.534	21.6
	Breed-Thoroughbred	0.118	4.98	0.542	45.8
EqCoPV	Age	0.502	1.0383	0.9303	1.159
	Country of foaling-Korea	0.678	0.748	0.19	2.95
	Clinical disease-Colic	0.025 *	3.79	1.11	12.9
	Purpose-Racing	0.524	1.54	0.402	5.94
	Purpose-Breeding	0.848	0.857	0.177	4.16
	Breed-Thoroughbred	0.573	0.714	0.221	2.31

* *p* < 0.05, EqPV-CSF, equine parvovirus-cerebrospinal fluid; EqCoPV, eqcopivirus.

**Table 4:** Nucleotide (upper right) and amino acid (bottom left) similarities of the NS region in members of the genus *Copiparvovirus*.

	EqPV-CSF (Korea)	EqPV-CSF (USA)	EqCo-PV (Korea)	EqCo-PV-11 (USA)	EqCo-PV-8 (USA)	Sesa-Virus	BPV2	PPV4	RDCV	PPV6	Bosa-Virus	EqPV-H
EqPV-CSF (Korea)		97.9	56.5	57.1	58.0	48.0	50.1	46.5	46.4	44.9	48.1	46.8
EqPV-CSF (USA)	99.3		57.3	57.8	58.2	48.1	50.1	45.9	46.4	44.7	48.2	46.4
EqCoPV (Korea)	40.4	40.5		92.1	92.4	47.3	47.1	46.5	45.0	44.7	48.3	43.1
EqCoPV-11 (USA)	40.3	40.4	95.0		95.7	47.6	47.0	46.5	44.6	44.4	48.3	42.7
EqCoPV-8 (USA)	40.9	41.0	95.2	96.1		48.0	47.6	47.1	45.0	43.9	48.9	43.6
Sesavirus	28.1	28.3	25.1	25.3	25.6		45.3	45.1	42.5	44.6	45.0	44.5
BPV2	29.3	29.2	27.8	27.5	27.5	27.5		49.0	53.0	45.2	49.7	46.5
PPV4	30.4	30.5	32.1	31.1	31.3	28.5	35.6		48.4	57.7	47.5	46.9
RDCV	28.8	28.9	26.1	25.8	25.8	25.3	48.8	33.5		48.4	47.7	44.7
PPV6	29.2	29.4	29.9	29.7	29.5	28.1	33.5	57.5	33.2		47.4	46.5
Bosavirus	25.0	25.2	28.6	28.9	29.1	25.4	31.0	34.6	32.6	35.4		46.7
EqPV-H	28.5	28.3	26.7	26.6	26.6	28.4	35.2	34.6	30.1	34.6	32.4	

Abbreviations: BPV, bovine parvovirus; PPV, porcine parvovirus; RDCV, Roe deer copiparvovirus.

## Data Availability

The sequence reported in this study have been deposited in the GenBank database (accession no. OK422845 to OK422862).

## Supplementary Materials

The following are available online at https://www.mdpi.com/article/10.3390/vetsci8110268/s1, Figure S1. Phylogenetic analysis based on a partial NS gene (872 bp) of EqPV-CSF, Figure S2. Phylogenetic analysis based on a partial VP gene (479 bp) of EqCoPV, Table S1. Primer sets and PCR information for EqPV-CSF/EqCoPV detection, Table S2. Detailed information on clinical signs of horses in this study.
