# Supplementary material for: First Detection and Genetic Characterization of New Equine Parvovirus Species Circulating among Horses in Korea
Source: Vet Sci. 2021 Nov 7;8(11):268. doi: 10.3390/vetsci8110268 (PMC8621016; doi:10.3390/vetsci8110268)

Table S1. Primer sets and PCR information for EqPV-CSF/EqCoPV detection.

PCR	Primer Sets	Sequences (5' → 3')	Product Size, bp	Tm	No. PCR Cycles	References
EqPV-CSF detection (partial NS gene)	Forward	CAAATGGACCAATGCAAGCAG	996	50℃	40	[16]
	Reverse	ATGGTCCATAGTGACCAGTGTC				
EqCoPV detection (partial VP gene)	Forward	GTGGACAGCCGAAGAGTGGA	522	50℃	40	[1]
	Reverse	AGTCACTCGGCCATGGTGTT				
EqPV-CSF (complete CDS)	EqPV 1F	ATACCCCAACAGAAACAAGG	1145	56℃	40	MZ100069.1
	EqPV 1145R	TGTCCTTCATCCCACCATCC				
	EqPV 940F	GGAATGCTATTCTGGCTATGGTC	1153	56℃	40	
	EqPV 2092R	GTCTCTGGTGCTTTCTGTGC				
	EqPV 1922F	ACCAGGACCAGACGCATATT	1148	56℃	40	
	EqPV 3069R	ACGCATCCTGTGAAGGTACA				
	EqPV 2724F	AGTTGTGAGAAACGCCTGCT	1551	56℃	40	
	EqPV 4274R	TGCTGTGTCTTGGGTTCTTG				
	EqPV 3939F	GACAATCCATGGGCCAAACA	1111	56℃	40	
	EqPV 5049R	CGGAGGTGAGGCTACATTCC				
EqCoPV (complete CDS)	EqCo 26F	GCGTTGCAACAACCACAAAA	1236	56℃	40	MN181468.1
	EqCo 1261R	TAGTGCTCCTTCATCCCACC				
	EqCo 617F	CTTCGATCCTGGACAACCAT	1637	56℃	40	
	EqCo 2253R	TAGCAGCCCTCCCAATGTAG				
	EqCo 2027F	GGTCACAAAAAGGCACTGGT	1262	56℃	40	
	EqCo 3288R	GCTAAGCCTCCAAGTTCAGC				
	EqCo 2910F	CAACGATACCGTGTGGACAG	1311	56℃	40	
	EqCo 4220R	AGTAGTCCCCGTCCCTATGT				
	EqCo 3981F	TACCAATGGAACCCCTCACA	1101	56℃	40	
	EqCo 5081R	GTCTGTCTCTCATGGGCCAT				
	EqCo 4810F	AATGGTACCAACACCAGGAC	289	60℃	40	
	EqCo 5098F	TTATCTCATTCTTCTGGGTCTG				

Abbreviations: bp, base pair; CDS, coding sequences; PCR, polymerase chain reaction; NS, non-structural; Tm, melting temperature.

Table S2. Detailed information on clinical signs of horses in this study.

Clinical Signs	Number of Horses
Colic	41
Osteochondrosis	8
Diarrhea	4
Dystocia	3
Pneumonia	3
Wound	3
Anorexia	2
Cachexia	2
Cellulitis	2
Equine Neonatal Maladjustment Syndrome	2
Hyperthermia	2
Limb deformity	2
Respiratory noise	2
Sesamoid bone fracture	2
Arthritis	1
Castration	1
Corneal ulcer	1
Dermatitis	1
Esophageal stricture	1
Guttural pouch empyema	1
Lameness	1
Laminitis	1
Strangles	1
Suspensory ligament rupture	1

Figure S1. Phylogenetic analysis based on a partial NS gene (872 bp) of EqPV-CSF. Newly identified Korean isolates are indicated by black diamonds. The Korean isolates were analyzed together with other members of genus *Copiparvovirus*.

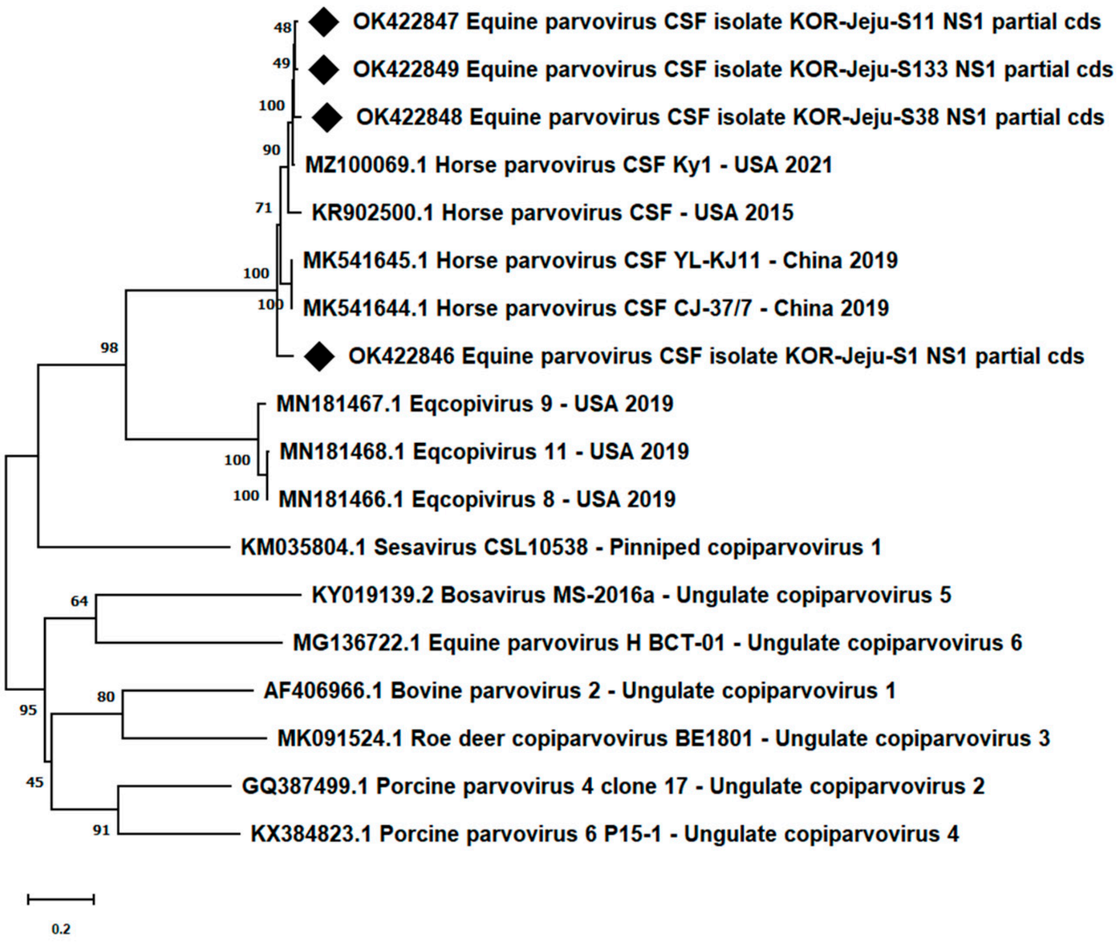

Figure S2. Phylogenetic analysis based on a partial VP gene (479 bp) of EqCoPV. Newly identified Korean isolates are indicated by black circles. The Korean isolates were analyzed together with other members of genus *Copiparvovirus*.

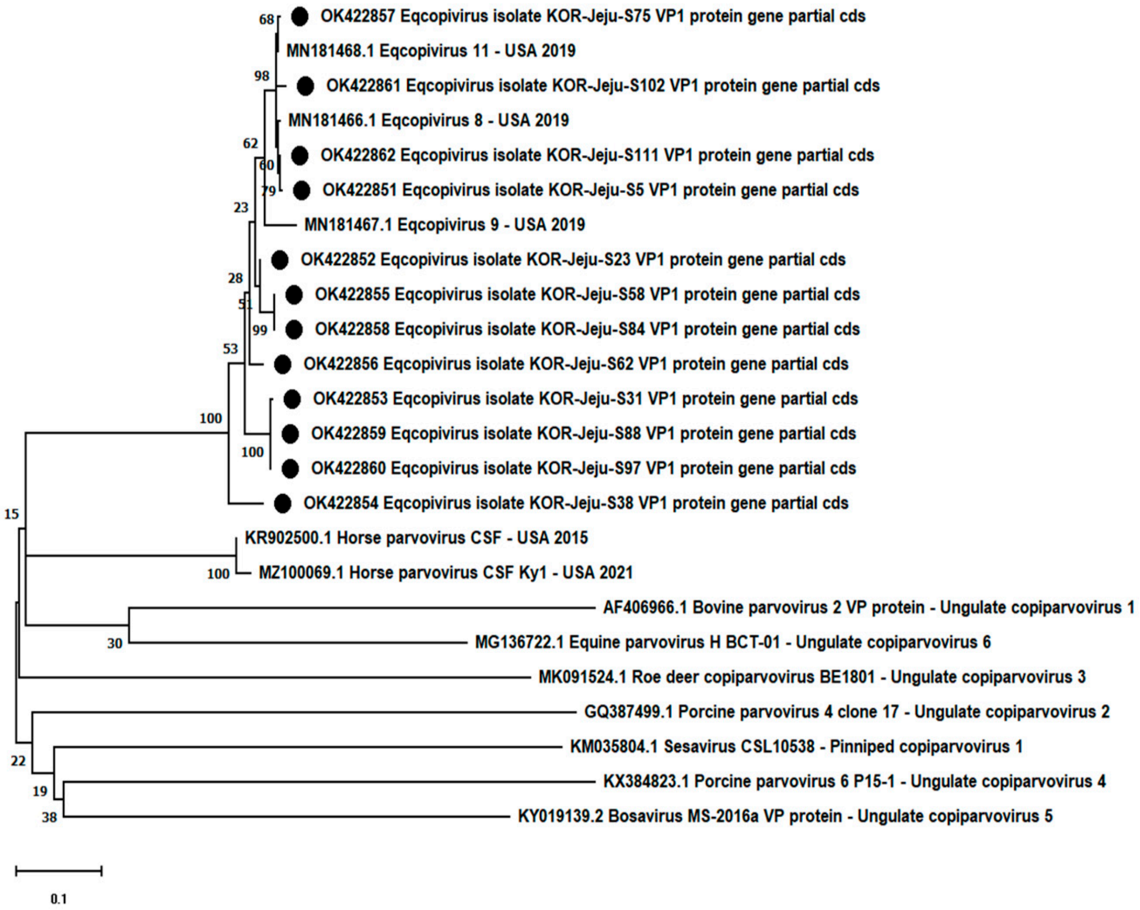

Supplement: Supplementary file 1 [file vetsci-08-00268-s001.zip › vetsci-1433722-supplementary.pdf]
